# Miscarriage following dengue virus 3 infection in the first six weeks of pregnancy of a dengue virus-naive traveller returning from Bali to Italy, April 2016

**DOI:** 10.2807/1560-7917.ES.2016.21.31.30308

**Published:** 2016-08-04

**Authors:** Maurizio Zavattoni, Francesca Rovida, Giulia Campanini, Elena Percivalle, Antonella Sarasini, Graziella Cristini, Lina Rachele Tomasoni, Francesco Castelli, Fausto Baldanti

**Affiliations:** 1 Molecular Virology Unit, Microbiology and Virology Department, Fondazione IRCCS Policlinico San Matteo, Pavia, Italy; 2 These authors contributed equally to this work; 3 Department of Infectious Diseases, Spedali Civili General Hospital, Brescia, Italy; 4 University Department of Infectious and Tropical Diseases, University of Brescia and Spedali Civili General Hospital, Brescia, Italy; 5 Department of Clinical, Surgical, Diagnostic and Pediatric Sciences, University of Pavia, Pavia, Italy

**Keywords:** dengue virus 3, pregnancy, travel

## Abstract

We report miscarriage following dengue virus (DENV)-3 infection in a pregnant woman returning from Bali to Italy in April 2016. On her arrival, the woman had fever, rash, asthenia and headache. DENV RNA was detected in plasma and urine samples collected the following day. Six days after symptom onset, she had a miscarriage. DENV RNA was detected in fetal material, but in utero fetal infection cannot be demonstrated due to possible contamination by maternal blood.

## Case description

A woman in her 30s returned from Bali to Italy in early April 2016. The day she left Bali and arrived in Italy, she became ill with fever (> 38.5 °C), rash, asthenia and headache, which lasted for a further five days. She had had her last menstrual period mid-February and discovered she was seven-weeks pregnant on her return from Bali, having performed an off-the-shelf pregnancy test as soon as she landed in Italy. The day of her arrival (during the first 24 hours of fever), she presented at a hospital emergency department in Brescia (Lombardy region), where ultrasonographic examination confirmed she was pregnant. The size and cardiac activity of the embryo was normal. All haematochemical tests of the woman were normal (including white blood cell and platelet count), a rapid diagnostic test (BinaxNOW Malaria, Alere, Scarborough, United States) for malaria was negative, TORCH assays were negative for cytomegalovirus and toxoplasmosis, while she was immune to herpes simplex-1 virus and rubella virus, and she was discharged. Because of persistence of her symptoms, including a high temperature (> 38.5 °C), she returned the following day: she was mildly neutropenic but not platelet depleted. Ultrasound confirmed a live embryo. Blood and urine samples were collected and referred to the regional reference laboratory (Fondazione IRCCS Policlinico San Matteo in Pavia) to investigate potential arbovirus infections. Three days later, her platelet level started to fall, with the lowest count (30,000/μL; norm: 130,000–400,000/μL) recorded three days after that. 

Three days after arriving in Italy, the woman’s spouse, who had also been travelling in Bali, reported similar symptoms.

## Laboratory findings

The diagnostic assessment included the following: (i) detection of dengue virus (DENV) 1–4 IgM and IgG antibodies in serum samples (using dengue virus IgM Capture DxSelect and dengue virus IgG DxSelect, Focus Diagnostics, United States), as well as detection of Zika virus (ZIKV) IgM and IgG antibodies (Anti-Zika virus ELISA (IgM) and Anti-Zika virus ELISA (IgG), Euroimmun, Germany); (ii) serology results were confirmed by neutralisation assay [1]; (iii) detection of DENV NS1 antigen in serum samples (dengue NS1 Ag STRIP, BIO RAD, France); (iv) detection of DENV RNA and ZIKV RNA in plasma and urine samples using a pan-flavivirus hemi-nested reverse transcription(RT)- polymerase chain reaction (PCR) targeting a conserved region of the NS5 gene [2] as well as virus-specific real-time RT-PCRs, targeting a conserved region in the 3’ untranslated region of DENV 1–4 [3] and a portion of the envelope protein gene of ZIKV [4]; and (v) sequencing of positive pan-flavivirus amplicons.

DENV infection was diagnosed in the woman and her spouse, while ZIKV infection was ruled out.

Two days after symptom onset, the woman’s serum tested negative for DENV IgG and IgM, while NS1 antigenaemia and high levels of DENV RNA (7.0 × 10^8^ copies/mL) were detected in her plasma (Table). DENV RNA was detected in her urine (1.0 × 10^3^ copies/mL).

**Table:** Virological results in two dengue virus-infected patients returning from Bali to Italy, April 2016

Patient	Days after symptom onset samples taken	IgG	IgM	DENV-3 NT Ab	NS1 antigen	Dengue virus-specific real-time RT-PCR Number of copies/mL			Pan-flavivirus RT-PCR		
						Plasma	Urine	Fetal material^b^	Plasma	Urine	Fetal material^b^
Woman^a^	+ 2 (7 WOP)	Neg	Neg	< 1:10	Pos	7.0 × 10^8^	1.0 × 10^3^	NA	Pos	Pos	NA
	+ 9 (8 WOP)	ND	ND	ND	ND	ND	ND	3.9 × 10^3^	ND	ND	Pos
	+ 12	Neg	Pos	1:80	Neg	20	1.6 × 10^3^	NA	Pos	Pos	NA
	+ 24	Pos	Pos	1:160	Neg	Neg	Neg	NA	Pos	Pos	NA
Woman’s spouse	+ 5	Neg	Pos	1:20	Pos	Neg	6.7 × 10^2^	NA	Pos	Pos	NA
	+ 21	Pos	Pos	1:160	Neg	Neg	Neg	NA	Pos	Pos	NA

DENV: dengue virus; NA: not applicable; ND: not done; Neg: negative; NT Ab: neutralising antibody titre; Pos: positive; RT-PCR: reverse transcription-polymerase chain reaction; WOP; weeks of pregnancy.

^a^ The woman had a miscarriage six days after symptom onset.

^b^ Tested after miscarriage.

Sequencing of amplicons from the woman’s plasma and urine confirmed infection by a DENV-3 serotype (GenBank accession number KX583642-KX583643). Interestingly, the DENV RNA load in her plasma was the highest observed at our institution for several years (the median value of 10 sequential recent imported DENV cases is reported for comparison: 3.1 × 10^4^ DENV RNA copies/mL; range: 8.8 × 10^2^ to 5.4 × 10^6^ copies/mL). In addition, DENV was isolated from the plasma sample. 

Six days after symptom onset, the woman had a miscarriage: there was no fetal cardiac activity. Three days later, she underwent surgical uterine evacuation and DENV RNA (3.9 × 10^3^ copies/mL) was detected in the fetal material (GenBank accession number KX583644).

A dramatic reduction of DENV RNA load in the woman’s plasma was observed six days after the miscarriage (20 copies/mL), while NS1 Ag was negative. At this time (12 days after symptom onset), a plasma sample was positive for DENV IgM, while IgG tests were still negative. DENV real-time RT-PCR to detect DENV RNA in plasma and urine was negative 24 days after symptom onset, whereas it was still positive by pan-flavivirus hemi-nested RT-PCR. DENV IgG seroconversion was observed at that point (Table).

Results from virological and serological tests of her spouse’s samples were somewhat different. Five days after symptom onset, DENV NS1 Ag was positive but DENV RNA in plasma was detected only by pan-flavivirus hemi-nested RT-PCR. In contrast, DENV RNA was detected in urine by both molecular assays. At that time, the sample was DENV IgM positive. Tests 21 days after symptom onset showed that NS1 Ag was negative in plasma and urine, as was DENV RNA using specific real-time RT-PCR, while both samples were positive using the pan-flavivirus hemi-nested RT-PCR. IgG seroconversion was seen at that point (Table).

## Background

The recently reported clusters of microcephaly and other birth defects caused by ZIKV infection in South America [5] have prompted European countries to be on alert for arthropod-born infectious disease risks, especially regarding pregnant travellers and their sexual partners. While chikungunya and West Nile virus infections have not proved to be an increased risk of preterm delivery, miscarriage or low birth weight [6-8], maternal and fetal consequences of DENV infection during pregnancy can be severe [9-12]. In tropical and subtropical regions, four serotypes (DENV 1–4) may be endemic in the same human population and clinical manifestations may range from mild fever in primary infections to haemorrhagic syndromes in reinfections by a different serotype [13]. Other factors, such as high viraemia titre and increased dengue virus-specific serotype replication have been postulated in the pathogenesis of severe disease [14]. In pregnant women living in endemic regions, dengue fever and severe dengue can develop; low platelet counts have been seen in both primary and secondary infections [12]. Fetal death, premature birth and low birth weight, as well as vertical transmission at term causing neonatal thrombocytopenia, have been recorded [10]. In contrast, however, the impact of DENV infection on pregnancy outcome in dengue immunologically naive travellers and the relationship between peak viraemia and pregnancy outcome remain unexplored.

## Discussion

DENV infection during the first trimester of pregnancy does not reveal a significant risk of vertical transmission of the virus [10]. However, since women in early pregnancy may not be hospitalised, the frequency of vertical transmission remains difficult to estimate [15-18]. 

The case described in our report chronologically links an acute DENV infection in the first weeks of pregnancy to an unfavourable outcome. It should be borne in mind, however, that one in five of all recognised pregnancies end in miscarriage [19] and maternal hyperthermia is recognised as an independent risk factor for miscarriage [20]. 

Although DENV RNA was detected in the fetal material tested after the miscarriage, in utero fetal infection cannot be demonstrated due to possible contamination with maternal blood. As fetal death followed the onset of DENV infection, adverse fetal outcome due to possible effects of high maternal viraemia on placental endothelial function cannot be excluded [14,21].

In the global alertness for ZIKV-induced microcephaly [5], DENV infection might represent an additional and underestimated risk factor for pregnancy.
